# When does a generalized Boolean quasiring become a Boolean ring?

**DOI:** 10.1007/s00500-017-2983-y

**Published:** 2017-12-19

**Authors:** Ivan Chajda, Helmut Länger

**Affiliations:** 1 Department of Algebra and Geometry, Faculty of Science, Palacký University Olomouc, 17. listopadu 12, 771 46 Olomouc, Czech Republic; 2 Faculty of Mathematics and Geoinformation, Institute of Discrete Mathematics and Geometry, TU Wien, Wiedner Hauptstraße 8-10, 1040 Vienna, Austria

**Keywords:** Generalized Boolean quasiring, Boolean ring with unit, Lattice with an antitone involution, Axiomatic quantum mechanics

## Abstract

Generalized Boolean quasirings are ring-like structures used as algebraic models in the foundations of axiomatic quantum mechanics. The quantum mechanical system corresponding to such a quasiring turns out to be a classical one if and only if this quasiring is a Boolean ring with unit. We characterize this situation by a single identity.

The classical approach to the foundations of axiomatic quantum mechanics was introduced by Mackey (2004). It was modified by D. Dorninger, G. Eigenthaler, M. Mączyński and both authors, see Chajda (1996), Chajda and Eigenthaler (1998), Chajda and Länger (2007), Chajda et al. (2004), Dorninger (1998), Dorninger et al. (1997a, b, 1999, 2000, 2001a, b), Länger (1998), Länger and Mączyński (2005). (For concepts concerning lattice theory we refer the reader to the monograph (Grätzer 2011).) They introduced several so-called ring-like structures used for modelling quantum logics. These ring-like structures are called Boolean quasirings, generalized Boolean quasirings and orthopseudorings (Chajda 1996; Chajda and Eigenthaler 1998) and their mutual relations were investigated by Chajda and Eigenthaler (1998).

It is known that for classical physics the corresponding logical structure are Boolean algebras which are in one-to-one correspondence to Boolean rings with unit. Hence it is a natural question to find the difference between Boolean rings with unit and the mentioned ring-like structures. In the following we present an identity which has the property that when added to the identities of a uniquely representable generalized Boolean quasiring converts this ring-like structure into a Boolean ring with unit.

Recall that a *Boolean ring with unit* is a unitary ring $\mathbf {R}=(R,+,\cdot ,0,1)$ that is idempotent, i.e. the identity $xx\approx x$ holds. (Here and in the following identities are written by using the symbol “$\approx$” which means that the corresponding identity holds for arbitrary elements of the considered algebra(s).) It is well-known that every Boolean ring is commutative and of characteristic 2, i.e. the identity $x+x\approx 0$ holds. The notion of a generalized Boolean quasiring was introduced in Dorninger et al. (1997a) as follows:

## Definition 1

A *generalized Boolean quasiring* is an algebra $\mathbf {R}=(R,+,\cdot ,0,1)$ of type (2, 2, 0, 0) satisfying the following identities:

1 $$\begin{aligned} x+y\approx & {} y+x \end{aligned}$$

2 $$\begin{aligned} x+0\approx & {} x \end{aligned}$$

3 $$\begin{aligned} (xy)z\approx & {} x(yz) \end{aligned}$$

4 $$\begin{aligned} xy\approx & {} yx \end{aligned}$$

5 $$\begin{aligned} xx\approx & {} x \end{aligned}$$

6 $$\begin{aligned} x0\approx & {} 0 \end{aligned}$$

7 $$\begin{aligned} x1\approx & {} x \end{aligned}$$

8 $$\begin{aligned} 1+(1+xy)(1+y)\approx & {} y \end{aligned}$$

The algebra $\mathbf {R}$ is called *uniquely representable* (cf. Dorninger 1998) if it satisfies the identity

9 $$\begin{aligned} (1+(1+x)(1+y))(1+xy)\approx x+y \end{aligned}$$

and a *Boolean quasiring* (cf. Dorninger et al. 1997a) if it satisfies identity (9) as well as the following identity:

10 $$\begin{aligned} x+x\approx 0. \end{aligned}$$

## Remark 2

Identity (1) follows from identities (4) and (9). This means that commutativity of addition follows from unique representability of the corresponding generalized Boolean quasiring.

In Dorninger et al. (1997a) the following result was proved:

## Theorem 3

A Boolean quasiring $\mathbf {R}=(R,+,\cdot ,0,1)$ is a Boolean ring with unit if and only if it satisfies the identity

11 $$\begin{aligned} x(1+y)\approx x+xy. \end{aligned}$$

Recall that a *bounded lattice with an antitone involution* is an algebra $\mathbf {L}=(L,\vee ,\wedge ,',0,1)$ such that $(L,\vee ,\wedge ,0,1)$ is a bounded lattice and the following identities are satisfied:

$$\begin{aligned} (x\vee y)'\approx & {} x'\wedge y', \\ (x\wedge y)'\approx & {} x'\vee y', \\ (x')'\approx & {} x. \end{aligned}$$

The following well-known result (cf. Dorninger et al. 1997a) shows that every uniquely representable generalized Boolean quasiring can be assigned to a bounded lattice with an antitone involution and conversely.

## Proposition 4

The formulas

$$\begin{aligned} x\vee y:=1+(1+x)(1+y), x\wedge y:=xy\text { and }x':=1+x \end{aligned}$$

and

$$\begin{aligned} x+y:=(x\vee y)\wedge (x'\vee y')\text { and }xy:=x\wedge y \end{aligned}$$

induce a one-to-one correspondence between uniquely representable generalized Boolean quasirings and bounded lattices with an antitone involution.

The aim of the present paper is to generalize Theorem 3 for generalized Boolean quasirings as follows:

## Theorem 5

A uniquely representable generalized Boolean quasiring $\mathbf {R}=(R,+,\cdot ,0,1)$ is a Boolean ring with unit if and only if it satisfies the identity

12 $$\begin{aligned} x(1+xy)\approx x(1+y). \end{aligned}$$

## Remark 6

Identities (3), (5) and (11) together imply (12). This means that Theorem 5 is in fact stronger than Theorem 3.

We start with some easy observations:

## Lemma 7

(i) Identities (5) and (8) together imply the identities
  13 $$\begin{aligned} 1+(1+x)\approx x \end{aligned}$$
  and
  14 $$\begin{aligned} (1+xy)(1+y)\approx 1+y. \end{aligned}$$
(ii) Identities (2), (5) and (8) together imply the identity $1+1\approx 0$.
(iii) Identities (2), (5), (6), (7), (8) and (12) together imply the identity $x(1+x)\approx 0$.

## Proof

(i) Identities (5) and (8) together imply $1+(1+x)\approx 1+(1+x)(1+x)\approx 1+(1+xx)(1+x)\approx x$ and hence $(1+xy)(1+y)\approx 1+(1+(1+xy)(1+y))\approx 1+y$.
(ii) Identity (2) and (i) together imply $1+1\approx 1+(1+0)\approx 0$.
(iii) Identities (7), (12) and (6) and (ii) together imply $x(1+x)\approx x(1+x1)\approx x(1+1)\approx x0\approx 0$.

$$\square $$

The most important property that discerns Boolean rings with unit from generalized Boolean quasirings is distributivity. In order to prove this property the following result will be useful:

## Lemma 8

Every generalized Boolean quasiring $\mathbf {R}=(R,+,\cdot ,0,1)$ satisfying identity (12) also satisfies the identity $(1+(1+x)(1+y))z\approx 1+(1+xz)(1+yz)$.

## Proof

Using mainly identities (12), (13) and (14) we obtain

$$\begin{aligned}&(1+(1+x)(1+y))z \\&\quad \approx (1+(1+x)(1+y)z)z\approx (1+(1+x)(1+yz)z)z\\&\quad \approx (1+\,(1+x)(1+yz))z \\&\quad \approx 1+(1+(1+(1+x)(1+yz))z) \\&\quad \approx 1+(1+(1+(1+x)(1+yz))z)(1+(1\\&\qquad +\,(1+x)(1+yz))yz) \\&\quad \approx 1+(1+(1+(1+x)(1+yz))z)(1+(1\\&\qquad +\,(1+x)(1+yz))(1+(1+yz))) \\&\quad \approx 1+(1+(1+(1+x)(1+yz))z)(1+(1+(1+yz))) \\&\quad \approx 1+(1+(1+(1+x)(1+yz))z)(1+yz) \\&\quad \approx 1+(1+(1+(1+x)(1+yz))z(1+yz))(1+yz)\\&\quad \approx 1+(1+(1+(1+x))z(1+yz))(1+yz)\\&\quad \approx 1+(1+\,xz(1+yz))(1+yz)\\&\quad \approx 1+(1+xz)(1+yz). \end{aligned}$$

$$\square $$

With the previous results in hand, we obtain now a simple proof of Theorem 5.

## Proof of Theorem 5

Let $\mathbf {R}=(R,+,\cdot ,0,1)$ be a uniquely representable generalized Boolean quasiring satisfying identity (12) and $\mathbf {L}=(R,\vee ,\wedge ,',0,1)$ denote the corresponding bounded lattice with an antitone involution. According to Lemma 7, $\mathbf {L}$ satisfies the identity $x\wedge x'\approx 0$ and, according to Lemma 8, $\mathbf {L}$ is distributive. Hence $\mathbf {L}$ is a Boolean algebra and therefore $\mathbf {R}$ a Boolean ring with unit. The rest of the proof is clear. $\square$
